# Arginines Plasma Concentration and Oxidative Stress in Mild to Moderate COPD

**DOI:** 10.1371/journal.pone.0160237

**Published:** 2016-08-01

**Authors:** Angelo Zinellu, Alessandro Giuseppe Fois, Salvatore Sotgia, Elisabetta Sotgiu, Elisabetta Zinellu, Fabiana Bifulco, Arduino A Mangoni, Pietro Pirina, Ciriaco Carru

**Affiliations:** 1 Department of Biomedical Sciences, University of Sassari, Sassari, Italy; 2 Department of Clinical and Experimental Medicine, University of Sassari, Sassari, Italy; 3 Department of Clinical Pharmacology, School of Medicine, Flinders University, Adelaide, Australia; 4 Quality Control Unit, University Hospital Sassari (AOU), Sassari, Italy; Research Center Borstel, GERMANY

## Abstract

**Background:**

Elevated plasma concentrations of the endogenous nitric oxide synthase (NOS) inhibitor asymmetric dimethylarginine (ADMA) have been observed in respiratory conditions such as asthma and cystic fibrosis. Since oxidative stress has been shown to increase the activity of arginine methylating enzymes, hence increased ADMA synthesis, and to reduce ADMA degrading enzymes, hence increased ADMA concentrations, we assessed methylated arginines concentrations in chronic obstructive pulmonary disease (COPD), a disease characterized by increased oxidative stress.

**Methods:**

Plasma arginine, ADMA and symmetric dimethylarginine (SDMA), oxidative stress markers (thiobarbituric acid reactive substances, TBARS, and plasma proteins SH, PSH) and antioxidants (taurine and paraoxonase 1, PON1, activity) were measured in 43 COPD patients with mild (n = 29) or moderate (n = 14) disease and 43 age- and sex-matched controls.

**Results:**

TBARS significantly increased with COPD presence and severity (median 2.93 *vs* 3.18 *vs* 3.64 μmol/L, respectively in controls, mild and moderate group, p<0.0001 by ANOVA) whereas PSH decreased (6.69±1.15 vs 6.04±0.85 vs 5.33±0.96 μmol/gr prot, p<0.0001 by ANOVA). Increased ADMA/arginine ratio, primarily due to reduced arginine concentrations, was also observed with COPD presence and severity (median 0.0067 vs 0.0075 vs 0.0100, p<0.0001 by ANOVA). In multiple logistic regression analysis, only TBARS (OR 0.44, 95% CI 0.25–0.77; p = 0.0045) and ADMA/Arginine ratio (OR 1.72, 95% CI 2.27–13.05; p = 0.02) were independently associated with COPD severity.

**Conclusion:**

COPD presence and severity are associated with increased oxidative stress and alterations in arginine metabolism. The reduced arginine concentrations in COPD may offer a new target for therapeutic interventions increasing arginine availability.

## Introduction

Chronic Obstructive Pulmonary Disease is a common respiratory condition characterized by progressive airflow limitation, persistent productive cough, mucous plugging and dyspnea [1–2]. Elevated concentrations of oxidative stress (OS) markers are commonly observed in this group [3–5]. The reduced ability of cellular antioxidant defenses to fully inactivate the reactive oxygen species (ROS) is a hallmark of OS. As a result, there is a functional impairment of several important biomolecules as lipids, proteins or nucleic acids, which can compromise cell health and viability. OS induces a variety of cellular responses through generation of secondary reactive species, leading to cell death by necrosis or apoptosis and, consequently, disease onset and progression. Moreover, activity of several enzymes can be influenced by redox regulation [6], including enzymes involved in the formation and degradation of asymmetric dimethylarginine (ADMA), such as protein arginine N-methyltransferases (PRMTs) and dimethylarginine dimethylaminohydrolase (DDAH) [7–8]. ADMA is an effective endogenous inhibitor of nitric oxide synthase (NOS). Its accumulation has been reported in renal failure [9–10], cardiovascular disease [11–12] and, only recently, lung disease [13–14]. ADMA synthesis is catalysed by PRMTs through the addition of one or two methyl groups to the terminal nitrogen atom of protein arginine. Human PRMTs are classified on the basis of their specific catalytic activities in type I and type II. In the first step, both enzymes catalyse monomethylarginine formation. During the second step type I enzymes produce ADMA, whereas type II enzymes lead to the formation of SDMA [15]. During proteolysis, ADMA and SDMA are released into the cytosol where free ADMA, but not free SDMA, is further degraded to citrulline and dimethylamine by DDAH15. Studies have shown that PRMT1 RNA or protein expression is increased, and DDAH activity is decreased, under OS stimuli [16–18]. While OS is well characterized, little information is available on methylated arginine concentrations in COPD. Available data principally focus on arginine and methylated arginines in sputum or exhaled breath condensate [19–22], while only one report describes plasma concentrations in COPD subjects [22]. Therefore, we tested the hypothesis that a) methylated arginines are associated with COPD presence and severity and b) such alterations are associated with OS markers (thiobarbituric acid reactive substances and Proteins–SH) and antioxidants (taurine and paraoxonase 1 activity).

## Methods

### Subjects

Forty-three consecutive COPD patients (29 mild and 14 moderate) without a previous diagnosis of COPD, were enrolled from the Respiratory Unit of the University of Sassari. Each patient underwent physical examination, chest radiographs, routine blood tests and respiratory function tests. The latter included forced expiratory volume in 1 sec (FEV1), forced vital capacity, and FEV_1_/FVC ratio. A structured questionnaire was administered to obtain demographic and clinical information including age, sex, body mass index (BMI) and smoking status. No patient was treated with long-acting muscarinic antagonists, long or short acting β-agonists at the time of the assessments. Moreover, no patient received inhaled corticosteroids within four weeks prior to the study. COPD patients with significant symptom deterioration within the last three months, indicative of disease exacerbation, were excluded.

COPD diagnosis and severity were assessed according to physical examination, spirometric results,

smoking history and respiratory symptoms based on the Global Initiative for Chronic Obstructive Lung Disease criteria [23]. In particular, classification of COPD severity was based on spirometric values reported in Table 1.

**Table 1:** Classification of COPD severity on the basis of spirometry values.

Severity of obstruction	Post bronchodilator FEV_1_/FVC	FEV_1_% PRED
MILD COPD	< 0.7	> 80%
MODERATE COPD	< 0.7	50–80%
SEVERE COPD	< 0.7	30–50%
VERY SEVERE	< 0.7	< 30%

A group of 43 age and sex-matched healthy controls was also included in the study. Exclusion criteria included the presence of concomitant inflammatory disease such as autoimmune disorders and infections, liver, kidney, heart disease and cancer.

This study was approved by the Institutional Local Ethics Committee (Azienda Sanitaria Locale n°1 di Sassari (Italy) (prot. 2175/CE del 21/04/2015), and was in accordance with the principles of Declaration of Helsinki. All subjects provided written informed consent.

### Biochemical analysis

Arginine, ADMA, SDMA and taurine were determined by capillary electrophoresis UV detection as previously described [24–25]. As inadequate precision of the assay used for the analysis of ADMA may increase the chance of statistical type 2 errors in clinical studies and may also lead to a severe underestimation of the strength of the association between ADMA and other biochemical or clinical variables [26], we used a capillary electrophoresis method that give inter-assay CV between 2 and 3% for Arginine, ADMA and SDMA measurement.

TBARS were determined according to the method described by Esterbauer and Cheeseman [27]. TBARS methodology measures MDA and other aldehydes produced by lipid peroxidation induced by hydroxyl free radicals. Plasma was mixed with 10% trichloroacetic acid and 0.67% thiobarbituric acid and heated at 95°C in a thermoblock heater for 25 min. TBARS were determined by measuring the absorbance at 535 nm. A calibration curve was obtained using standard MDA and each curve point was subjected to the same treatment as that of the samples. Paraoxonase activity was determined by measuring the increase in absorbance at 412 nm (formation of 4-nitrophenol) using paraoxon (O,O diethyl-O-p-nitrophenyl phosphate) as a substrate [28]. Enzyme activity was calculated by using the molar extinction coefficient of 17,100 M^−1^cm^−1^ and one unit (U) of paraoxonase activity was defined as 1 nmoL of 4-nitrophenol formed per minute. Plasma PSH determination was performed by spectrophotometry with 5,5'-dithiobis-2-nitrobenzoic acid (DTNB) as titrating agent by measuring the absorbance of conjugate at 405 nm [29]. Concentration in samples was determined from a GSH standard curve.

### Statistical analysis

All results are expressed as mean values (mean ± SD) or median values (median and range). Variables distribution was assessed by the Kolmogorov-Simirnov test. Statistical differences between groups were compared using unpaired Student’s t-test or Mann-Whitney rank sum test, as appropriate. Correlation analysis between variables was performed by Pearson's correlation or Spearman’s correlation as appropriate. Multiple comparisons were performed by one-way ANOVA. Levene's test for equality of error variances was employed, while student-Newman-Keuls test for all pairwise comparisons was used. Non-normally distributed variables were log10-transformed prior to being used with parametric tests. Normal distribution of the residuals was checked to assess the goodness of fit of the transformations.

Logistic regression analysis with COPD absence vs. presence as dependent variable was conducted to determine associations between variables potentially involved in disease development. A further logistic regression analysis with mild or moderate condition as dependent variable was conducted to determine associations between COPD severity and variables potentially involved in disease progression.

Statistical analyses were performed using MedCalc for Windows, version 15.4 64 bit (MedCalc Software, Ostend, Belgium) and SPSS for Windows, version 14.0 32 bit (IBM Corporation; Armonk, NY, USA).

## Results

Table 2 describes the clinical and demographic characteristics of COPD patients and age- and sex-matched controls. FEV_1_ decreased significantly from 2.75±0.59 L in controls to 2.24±0.56 L in mild and 1.56±0.32 L in moderate COPD patients (p<0.001), whereas FVC was 3.40±0.73 L, 3.18±0.77 L and 2.44±0.54 L, respectively (p<0.001). FEV_1_/FVC ratio was 80.4±4.9% in controls, 70.2±3.12% in mild, and 64.8±7.9% in moderate COPD patients, respectively (p<0.001).

**Table 2:** Clinical, functional and biochemical parameters of healthy subjects and COPD patients.

Characteristics	Controls (n = 43)	Mild COPD (n = 29)	Moderate COPD (n = 14)	*p* value
Age (years)	73.4±6.9	75.4±4.8	73.4±7.7	NS
Sex (F/M)	9/34	7/22	2/12	NS
BMI (kg/m^2^)	26.4±3.6	27.4±3.4	27.4±4.5	NS
Current smokers	3 (7%)	2 (6.9%)	1 (7.1%)	NS
Never smoked	14 (32.6%)	8 (27.6%)	2 (14.2%)	NS
Ex smokers	26 (60.4%)	19 (65.5%)	11 (78.6%)	NS
FEV_1_ (L)	2.75±0.59	2.24±0.56***	1.56 ± 0.32***°°°	<0.001
FVC (L)	3.40±0.73	3.18±0.77	2.44±0.54***°°	<0.001
FEV1/FVC	80.8±4.9	70.2±3.1***	64.8±7.9***°°	<0.001
TBARS (μmol/L)	2.93 (2.46–3.23)	3.18 (2.50–3.54)	3.64 (3.16–4.38)**°	0.003
PSH (μmol/ g prot)	6.69±1.15	6.04±0.85	5.33±0.96***°	<0.001
PON1(U/L)	253 (147–340)	230 (154–376)	211(157–284)	NS
Taurine (μmol/L)	55.8 (47.7–72.1)	59.3 (49.0–76.8)	57.6 (50.8–75.3)	NS
Arginine (μmol/L)	79.8 (68.3–90.4)	70.4 (60.3–78.2)*	53.4 (41.4–59.8)***°°	<0.001
ADMA (μmol/L)	0.488 (0.454–0.544)	0.505 (0.432–0.588)	0.513 (0.412–0.625)	NS
SDMA (μmol/L)	0.460 (0.395–0.590)	0.513 (0.429–0.594)	0.485 (0.456–0.577)	NS
ADMA/arginine	0.0067 (0.0056–0.0077)	0.0075 (0.0053–0.0098)	0.0100 (0.0079–0.0117)***°°	<0.001
ADMA/SDMA	1.07 (0.80–1.28)	0.98 (0.81–1.31)	1.12 (0.86–1.25)	NS

*P<0.05

**p<0.01

***p<0.001 vs Controls;

°P<0.05

°°p<0.01

°°°p<0.001 vs mild COPD obtained by ANOVA (Student-Newman-Keuls test for all pairwise comparisons or Krustall-Wallis test as appropriate)

TBARS plasma concentrations increased significantly with COPD presence and severity (p<0.001 by ANOVA). In particular we found a significant difference between controls and patients with moderate COPD (median 2.93 vs 3.64 μmol/L, p<0.01) and between mild and moderate COPD patients (median 3.18 vs 3.64 μmol/L, p<0.05). By contrast, a significant decrease in plasma PSH concentrations was observed with COPD presence and severity (p<0.001). Multiple comparisons by ANOVA showed significant differences in PSH mean values between controls and moderate COPD (6.69±1.15 μmol/g prot *vs* 5.33±0.96 μmol/g prot, p<0.001) and between mild and moderate COPD (6.04±0.85 *vs* 5.33±0.96 μmol/g prot P<0.001). ADMA and SDMA plasma concentrations were not significant different between controls and COPD patients. By contrast, median arginine concentrations were progressively lower in controls (79.8 μmoL), mild (70.4 μmol/L) and moderate COPD patients (53.4 μmolL, P<0.001). As consequence also ADMA/arginine showed significant differences according to COPD presence and severity (p = 0.0001). Multiple comparisons demonstrated a significant difference between controls and patients with moderate COPD (median 0.0067 vs 0.0100, p<0.001) and between mild and moderate group (median 0.0075 vs 0.0010, p<0.05).

As reported in Table 3, univariate analysis in COPD patients showed that FEV1 was correlated with age (rho = -0.31, p = 0.043), PSH (rho = 0. 36, p = 0.016) and ADMA/arginine ratio (rho = -0.43, p = 0.0001). In controls FEV1 was correlated only with age (rho = -0.34; p = 0.036) and sex (rho = -0.55, p<0.0001). Table 4 report as, after adjusting for age, sex, BMI, smoking status, TBARS, PSH and ADMA/arginine ratio, sex (β = -0.44, p = 0.007), PSH (β = 0.33, p = 0.047), and ADMA/arginine ratio (β = -0.45, p = 0.005) were independently associated with FEV1 in COPD patients in regression analysis. In controls, only age (β = -0.38, p = 0.009) and sex (β = -0.68, p = <0.0001) were independently associated with FEV1.

**Table 3:** Linear regression analysis between FEV1 and some demographic and biochemical variables in controls and COPD patients.

	Controls (n = 43)		COPD (n = 43)	
	r *or* rho	p-value	r *or* rho	p-value
Age	-0.34	0.036	-0.31	0.043
Sex	-0.55	<0.0001	--	--
PSH	--	--	0.36	0.016
ADMA/Arginine	--	--	-0.43	<0.0001

**Table 4:** Multiple regression analysis with FEV1 as dependent variable and age, sex, BMI, smoking status, TBARS, PSH and ADMA/arginine ratio as independent variables, in controls and COPD patients.

	Controls (n = 43)		COPD (n = 43)	
	β	p-value	β	p-value
Age	-0.38	0.009	--	--
Sex	-0.68	<0.0001	-0.44	0.007
PSH	--	--	0.33	0.047
ADMA/Arginine	--	--	-0.45	0.005

When considering controls and COPD patients together, a negative relationship between PSH and ADMA/arginine ratio was also observed (rho = -0.23, p = 0.033). In multiple logistic regression analysis of the total population (COPD and controls), after adjusting for age, sex, BMI, smoking status, ADMA/arginine ratio, TBARS, PSH, PON and taurine, only PSH (OR 0.44, 95% CI 0.25–0.77; p = 0.004) and ADMA/Arginine ratio (OR 172, 95% CI 2.27–13,055; p = 0.02) were independently associated with presence of COPD.

Data of multiple logistic regression analysis performed on 43 COPD patients according to disease severity (mild vs. moderate), after adjusting for age, sex, BMI, smoking status, ADMA/arginine ratio, TBARS, PSH, PON and taurine are reported in Table 5. Lower plasma PSH and higher TBARS and ADMA/arginine ratio were independently associated with disease severity.

**Table 5:** Logistic regression analysis (including TBARS, PSH and ADMA/arginine ratio) showing ORs for moderate disease.

Factor	Moderate disease		p-value
	OR	95%CI	
TBARS	481 x10^12^	26–9x10^27^	0.030
PSH	0.0125	0.0003–0.4731	0.018
ADMA/arginine	49x10^6^	25–96x10^12^	0.016

## Discussion

Chronic obstructive pulmonary disease is a major and increasing global health problem. According to the World Health Organization COPD will become the third leading cause of death and the fifth leading cause of disability in the world by 2020 [30]. Despite increasing awareness, the pathogenesis of COPD has received relatively little attention from clinicians, researchers, and the pharmaceutical industry [31]. This is likely due because COPD is viewed as self-inflicted (by smoking) and also because the underlying disease process is generally considered to be irreversible. Consequently, there is a fundamental lack of knowledge about the cellular, molecular, and genetic mechanisms of this pathology. COPD is associated with a chronic inflammatory response, predominantly in small airways and lung parenchyma, which is characterized by an increase of activated neutrophils and macrophages and increased numbers of inflammatory mediators in the airways [32–33]. It has been proposed that in COPD the increased oxidant burden may not be adequately counterbalanced by the lung antioxidant systems, resulting in OS. Increased OS may be induced both directly, as a result of smoking, or indirectly by the increased release of reactive oxygen species from airspace inflammatory cells stimulated by noxious particles and gases. Analysis of cell profile in alveoli and small airways of COPD patients shows, in fact, an increase in several inflammatory cell types, including macrophages, T lymphocytes, B lymphocytes, and neutrophils [34]. These cells, once activated, can generate anion superoxide (O2·^-^) probably through reduced nicotinamide adenine dinucleotide phosphate oxidase pathway. In addition, the impaired ventilation may results in a decreased hemoglobin oxygen saturation level (hypoxemia), resulting in local tissue hypoxia [35]. Experimental evidence suggests that hypoxaemia enhances OS in COPD [36] and that the source of ROS production in hypoxia is likely to be the mitochondria at the respiratory chain level [37]. In support of these observations we found increased concentrations of TBARS (+13.2%) and reduced concentrations of PSH (-13.3%) in COPD subjects vs. controls, indicating the presence of significant OS. PSH assay, in fact, provide a measure of total protein sulfhydryl groups in plasma. The most representative -SH group in plasma is that of human serum albumin, due to its high concentrations. The Cys^34^ –SH group of HSA represents ~80% of all reduced thiols in human plasma. It is an important scavenger of reactive oxygen and nitrogen species in blood acting as an effective redox buffer in the vascular compartment [38]. When oxidative stress compromise this redox buffer system, ROS are free to attack lipids, thus yielding some products of lipid peroxidation as malondialdehyde, measurable by TBARS assay. Interestingly, simple linear regression suggests that in COPD patients, FEV_1_ is associated with age, PSH and ADMA/Arginine ratio, while in controls only age and sex were related to FEV_1_. These data, further supported by multiple linear regression analysis after correction for other important variables, confirms that increased OS is strictly linked to deterioration in lung function. Moreover, similarly to our previous observations in the general older population [39], we found a significant negative association between ADMA/arginine ratio and FEV_1_, suggesting a detrimental effect of arginine methylation on key lung functional parameters. This observation was further confirmed by multiple logistic regression analysis indicating that PSH and ADMA/arginine ratio were independently associated with COPD development. When analyzing the factors independently associated with COPD severity both OS markers, PSH and TBARS, and ADMA/arginine ratio showed significant associations. Arginine metabolism plays an important role in the maintenance of airways tone and function by production of nitric oxide via the NOS pathway [40]. Dysregulation of the competing enzymes has been shown to contribute to airway obstruction in asthma and in patients with cystic fibrosis [13–14,21]. Bode-Boger and coworkers recently proposed the calculation of the ADMA/arginine ratio as an index reflective of NOS imbalances activity caused by the accumulation of ADMA. As such, a “normal” ADMA/arginine ratio is in the range of 0.0044–0.0076 [40], consistent with our reported values in the control group, while COPD subjects had higher values in accordance with recent data reported by Aydin et al. [22]. In this last report, however, there is no information regarding the impact of disease severity on ADMA concentrations. We found that, when categorizing on the basis of disease progression, only moderate COPD patients had values above the normal range, whereas mild COPD patients had median values in the normal range, further supporting the hypothesis that ADMA and arginine could be involved primarily during disease worsening. Pending additional evidence from experimental and human studies, it is plausible to speculate that COPD disease exacerbation states are associated with further increases both in oxidative stress and in the ADMA/Arginine ratio. The mechanisms responsible for the reported imbalance between ADMA and arginine may be related to OS. The reduction in arginine concentrations observed in COPD patients is likely due to the well-known increase of arginase activity stimulated by OS [41]. Moreover, the increase of neutrophil numbers typical of COPD may contribute to arginine depletion since these cells constitutively express high levels of arginase I in azurophilic granules. These granules may be released in patients with COPD together with other constituents of the granules such as elastase [42]. It is also known that neutrophil numbers increase as COPD worsens [43]. This might further explain the further reduction of arginine concentrations observed in moderate vs. mild forms of disease.

Moreover, the activity of the enzymes involved in the formation and degradation of ADMA such as PRMTs and DDAH is regulated in a redox-sensitive fashion [16–18]. Studies in cultured endothelial cells have reported that the gene expression of PRMTs is increased by oxLDL in a concentration-dependent manner [7]. There is growing body of evidence that OS decreases the activity of the ADMA demethylating enzyme, DDAH [8]. The presence of a reactive cysteine residue (Cys^249^) in the active site of DDAH leads to diminished activity of the enzyme in presence of ROS. Thus, OS, through DDAH inhibition, PRMTs synthesis stimulation and arginase increase activity might be primarily responsible for an imbalance of ADMA/arginine ratio. This hypothesis is also supported by the significant negative correlation observed between ADMA/arginine ratio and PSH in the analyzed subjects. In our COPD cohort, ADMA/arginine ratio is altered mainly because of a reduction in arginine concentrations, even if a non significant increase in ADMA levels of about 3.5% has been found in all COPD patients with a rise of about 5.2% in moderate COPD. It will be interesting to evaluate if ADMA concentrations are further increased in patients with more severe symptoms (COPD stages 3 and 4).SDMA concentrations and ADMA/SDMA ratios were also similar in controls and COPD patients. Obtained ADMA/SDMA values were besides in accordance to that reported by Bulau et al [42].

Therefore, our data indicate that OS and ADMA/arginine ratios are related in COPD patients, confirming previous reports showing reduced PRMT RNA or protein expression, arginase and DDAH activity under OS stimuli [16–18, 43–44]. Both PRMT and DDAH are widely expressed in lung tissue. Recent evidence also suggest that methylarginine metabolism in the lung may significantly contribute to circulating ADMA and SDMA concentrations [42]. In particular, pulmonary DDAH-1 is actively involved in ADMA degradation while PRMT-I pulmonary expression is related to increased protein arginine methylation of the lung proteome. Moreover, as previously discussed, neutrophils significantly contribute to arginine decrease in the lung through the release of arginase I. Although no specific assessment of the expression and activity of these enzymes in lung tissue was performed in our study, it is plausible that the structural and functional lung alterations in COPD may lead to changes in arginine metabolites plasma concentration.

In conclusion our data, while confirming the role of OS and imbalanced arginine concentrations in COPD patients, show for the first time that increased ADMA/arginine ratio is independently associated with OS and COPD severity. Further studies, with a larger number of subjects covering all stages of COPD disease, are required to fully characterize the impact of arginine and ADMA on disease worsening.

## Abbreviations

ADMA: asymmetric dimethylarginine
COPD: chronic obstructive pulmonary disease
NOS: nitric oxide synthase
OS: oxidative stress
PON1: paraoxonase 1, PSH, Proteins–SH
SDMA: symmetric dimethylarginine
TBARS: thiobarbituric acid reactive substances

## References

[1] Vestbo J, Hurd SS, Agustí AG, Jones PW, Vogelmeier C, Anzueto A, et al Global strategy for the diagnosis, management, and prevention of chronic obstructive pulmonary disease: GOLD executive summary. Am J Respir Crit Care Med 2013; 187: 347–65. 10.1164/rccm.201204-0596PP 22878278

[2] Kim V, Criner GJ. Chronic bronchitis and chronic obstructive pulmonary disease. Am J Respir Crit Care Med 2013;187:228–37. 10.1164/rccm.201210-1843CI 23204254 PMC4951627

[3] Paredi P, Kharitonov SA, Leak D, Paredi P, Kharitonov SA, Leak D, et al Exhaled ethane, a marker of lipid peroxidation, is elevated in chronic obstructive pulmonary disease. Am J Respir Crit Care Med 2000;162:369–73. 10934055 10.1164/ajrccm.162.2.9909025

[4] Morrison D, Rahman I, Lannan S, MacNee W. Epithelial permeability, inflammation, and oxidant stress in air spaces of smokers. Am J Respir Crit Care Med 1999;159:473–9. 9927360 10.1164/ajrccm.159.2.9804080

[5] Montuschi P, Collins JV, Ciabattoni G, Lazzeri N, Corradi M, Kharitonov SA, et al Exhaled 8-isoprostane as an in vivo biomarker of lung oxidative stress in patients with COPD and healthy smokers. Am J Respir Crit Care Med 2000;162:1175–7. 10988150 10.1164/ajrccm.162.3.2001063

[6] Deponte M, Horst Lillig C. Enzymatic control of cysteinyl thiol switches in proteins. Biol Chem 2015;396:401–13. 10.1515/hsz-2014-0280 25581754

[7] Böger RH, Sydow K, Borlak J, Thum T, Lenzen H, Schubert B, et al LDL cholesterol upregulates synthesis of asymmetrical dimethylarginine in human endothelial cells: involvement of S-adenosylmethionine-dependent methyltransferases. Circ Res 2000;87:99–105. 10903992 10.1161/01.res.87.2.99

[8] Leiper J, Murray-Rust J, McDonald N, Vallance P. S-nitrosylation of dimethylarginine dimethylaminohydrolase regulates enzyme activity: further interactions between nitric oxide synthase and dimethylarginine dimethylaminohydrolase. Proc Natl Acad Sci USA 2002;99:13527–32. 12370443 10.1073/pnas.212269799 PMC129707

[9] Vallance P, Leone A, Calver A, Collier J, Moncada S. Accumulation of an endogenous inhibitor of nitric oxide synthesis in chronic renal failure. Lancet 1992; 339: 572–5. 1347093 10.1016/0140-6736(92)90865-z

[10] Schwedhelm E, Boger RH. The role of asymmetric and symmetric dimethylarginines in renal disease. Nat Rev Nephrol 2011; 7: 275–85. 10.1038/nrneph.2011.31 21445101

[11] Kurz K, Teerlink T, Sarcletti M, Weiss G, Zangerle R, Fuchs D. Plasma concentrations of the cardiovascular risk factor asymmetric dimethylarginine (ADMA) are increased in patients with HIV-1 infection and correlate with immune activation markers. Pharmacol Res 2009; 60: 508–14. 10.1016/j.phrs.2009.07.009 19651212

[12] Boger RH, Maas R, Schulze F, Schwedhelm E. Asymmetric dimethylarginine (ADMA) as a prospective marker of cardiovascular disease and mortality—An update on patient populations with a wide range of cardiovascular risk. Pharmacol Res 2009; 60: 481–7. 10.1016/j.phrs.2009.07.001 19596069

[13] Grasemann H, Al-Saleh S, Scott JA, Shehnaz D, Mehl A, Amin R, et al Asymmetric dimethylarginine contributes to airway nitric oxide deficiency in patients with cystic fibrosis. Am J Respir Crit Care Med 2011;183:1363–8. 10.1164/rccm.201012-1995OC 21278301

[14] Scott JA, Grasemann H. Asymmetric dimethylarginine: A disease marker for asthma? Chest 2013;144:367–368. 10.1378/chest.13-0480 23918098

[15] Vallance P, Leiper J. Cardiovascular biology of the asymmetric dimethylarginine: dimethylarginine dimethylaminohydrolase pathway. Arterioscler Thromb Vasc Biol 2004; 24:1023–30. 15105281 10.1161/01.ATV.0000128897.54893.26

[16] Jiang JL, Zhang XH, Li NS, Rang WQ, Feng-Ye, Hu CP, et al Probucol decreases asymmetrical dimethylarginine level by alternation of protein arginine methyltransferase I and dimethylarginine dimethylaminohydrolase activity. Cardiovasc drugs ther 2006; 20: 281–94 16897158 10.1007/s10557-006-9065-1

[17] Chen Y, Xu X, Sheng M, Zhang X, Gu Q, Zheng Z. PRMT-1 and DDAHs-induced ADMA upregulation is involved in ROS- and RAS-mediated diabetic retinopathy. Exp eye res 2009;89:1028–34 10.1016/j.exer.2009.09.004 19748504

[18] Tyagi N, Sedoris KC, Steed M, Ovechkin AV, Moshal KS, Tyagi SC. Mechanisms of homocysteine-induced oxidative stress. Am J Physiol Heart Circ Physiol 2005; 289:2649–56 10.1152/ajpheart.00548.2005 16085680

[19] Scott JA, Duongh M, Young AW, Subbarao P, Gauvreau GM, Grasemann H. Asymmetric dimethylarginine in chronic obstructive pulmonary disease (ADMA in COPD). Int J Mol Sci 2014;15:6062–71. 10.3390/ijms15046062 24727374 PMC4013615

[20] Carraro S, Giordano G, Piacentini G, Kantar A, Moser S, Cesca L, et al Asymmetric dimethylarginine in exhaled breath condensate and serum of children with asthma. Chest 2013;144:405–10. 10.1378/chest.12-2379 23412513

[21] Scott JA, North ML, Rafii M, Huang H, Pencharz P, Subbarao P, et al Asymmetric dimethylarginine is increased in asthma. Am J Respir Crit Care Med 2011;184:779–85. 10.1164/rccm.201011-1810OC 21719758

[22] Aydin M, Altintas N, Cem Mutlu L, Bilir B3, Oran M3, Tülübaş F1 Asymmetric dimethylarginine contributes to airway nitric oxide deficiency in patients with COPD. Clin Respir J. 2015; 10.1111/crj.12337 26076870

[23] Pauwels RA, Buist AS, Calverley PM, Jenkins CR, Hurd SS; and GOLD ScientificCommittee. Global strategy for the diagnosis, management, and prevention ofchronic obstructive pulmonary disease. NHLBI/WHO Global Initiative for ChronicObstructive Lung Disease (GOLD) Workshop summary. Am J Respir Crit Care Med 2001;163:1256–76. 11316667 10.1164/ajrccm.163.5.2101039

[24] Zinellu A, Sotgia S, Usai MF, Pintus G, Deiana L, Carru C. Improved method for plasma ADMA, SDMA, and arginine quantification by field-amplified sample injection capillary electrophoresis UV detection. Anal Bioanal Chem 2011;399:1815–21. 10.1007/s00216-010-4580-0 21181467

[25] Zinellu A, Sotgia S, Scanu B, Chessa R, Gaspa L, Franconi F, et al Taurine determination by capillary electrophoresis with laser-induced fluorescence detection: from clinical field to quality food applications. Amino Acids 2009;36:35–41. 10.1007/s00726-007-0022-5 18193477

[26] Teerlink T. Measurement of asymmetric dimethylarginine in plasma: methodological considerations and clinical relevance. Clin Chem Lab Med 2005; 43:1130–1138 16197310 10.1515/CCLM.2005.197

[27] Esterbauer H, Cheeseman KH. Determination of aldehydiclipid peroxidation products: malonaldehyde and 4-hydroxynonenal. Methods Enzymol 1990;186:407–421 2233308 10.1016/0076-6879(90)86134-h

[28] Gan KN, Smolen A, Eckerson HW, La Du BN. Purification of human serum paraoxonase/arylesterase. Evidence for one esterase catalyzing both activities. Drug Metab Dispos 1991;19:100–6. 1673382

[29] Ellman GL. Tissue sulfhydryl groups. Arch Biochem Biophys 1959;82:70–7. 13650640 10.1016/0003-9861(59)90090-6

[30] Lopez AD, Murray CC. The global burden of disease, 1990–2020. Nat Med 1998;4: 1241–3. 9809543 10.1038/3218

[31] Barnes PJ, Kleinert S. COPD-A neglected disease. Lancet 2004;364:564–5. 15313342 10.1016/S0140-6736(04)16866-9

[32] Keatings VM, Barnes PJ. Granulocyte activation markers in induced sputum:comparison between chronic obstructive pulmonary disease, asthma, andnormal subjects. Am J Respir Crit Care Med 1997;155:449–53. 9032177 10.1164/ajrccm.155.2.9032177

[33] Bhowmik A, Seemungal TA, Sapsford RJ, Wedzicha JA. Relation of sputum inflammatory markers to symptoms and lung function changes in COPD exacerbations. Thorax 2000;55:114–20. 10639527 10.1136/thorax.55.2.114 PMC1745686

[34] Retamales I, Elliott WM, Meshi B, Coxson HO, Pare PD, Sciurba FC, et al Amplification of inflammation in emphysema and its association with latent adenoviral infection. Am J Respir Crit Care Med 2001;164:469–73 11500352 10.1164/ajrccm.164.3.2007149

[35] Koechlin C, Maltais F, Saey D, Michaud A, LeBlanc P, Hayot M, et al Hypoxaemia enhances peripheral muscle oxidative stress in chronic obstructive pulmonary disease. Thorax 2005;60:834–41. 15964914 10.1136/thx.2004.037531 PMC1747208

[36] Hoppeler H, Vogt M, Weibel ER, Flück M. Response of skeletal muscle mitochondria to hypoxia. Exp Physiol 2003;881:109–19. 10.1113/eph8802513 12525860

[37] Chandel NS, Schumacker PT. Cellular oxygen sensing by mitochondria: old questions, new insight. J Appl Physiol 2000;88:1880–9 10797153 10.1152/jappl.2000.88.5.1880

[38] Rossi R, Giustarini D, Milzani A, Dalle-Donne I. Cysteinylation and homocysteinylation of plasma protein thiols during ageing of healthy human beings. J Cell Mol Med 2009;13:3131–40. 10.1111/j.1582-4934.2008.00417.x 18624771 PMC4516472

[39] McEvoy MA, Schofield PW, Smith WT, Agho K, Mangoni AA, Soiza RL, et al Serum methylarginines and spirometry-measured lung function in older adults. PLoS One 2013;8:e58390 10.1371/journal.pone.0058390 23690915 PMC3655195

[40] Bode-Boger SM, Scalera F, Ignarro LJ. The L-arginine paradox: importance of the L-arginine/asymmetrical dimethylarginine ratio. Pharmacol Ther 2007;114:295–306. 17482266 10.1016/j.pharmthera.2007.03.002

[41] Caldwell RB, Toque HA, Narayanan SP, Caldwell RW. Arginase: an old enzyme with new tricks. Trends Pharmacol Sci 2015;36:395–405 10.1016/j.tips.2015.03.006 25930708 PMC4461463

[42] Bulau P, Zakrzewicz D, Kitowska K, Leiper J, Gunther A, Grimminger F, et al Analysis of methylarginine metabolism in the cardiovascular system identifies the lung as a major source of ADMA. Am J Physiol Lung Cell Mol Physiol 2007;292:18–24. 10.1152/ajplung.00076.2006 16891395

[43] Renkema TE, Postma DS, Noordhoek JA, Sluiter HJ, Kauffman HF. In vitro release of neutrophil elastase, myeloperoxidase and beta-glucuronidase in patients with emphysema and healthy subjects. Eur Respir J 1991;4:1237–1244 1666565

[44] Hogg JC, Chu S, Utokaparch R, Woods R, Elliott WM, Buzatu L, et al The nature of small-airway obstruction in chronic obstructive pulmonary disease. N Engl J Med 2004;350:2645–2653 15215480 10.1056/NEJMoa032158

